# Anticancer Activity in Honeybee Propolis: Functional Insights to the Role of Caffeic Acid Phenethyl Ester and Its Complex With γ-Cyclodextrin

**DOI:** 10.1177/1534735417753545

**Published:** 2018-02-02

**Authors:** Yoshiyuki Ishida, Ran Gao, Navjot Shah, Priyanshu Bhargava, Takahiro Furune, Sunil C. Kaul, Keiji Terao, Renu Wadhwa

**Affiliations:** 1 CycloChem Co, Ltd, Chuo-ku, Kobe, Japan; 2 National Institute of Advanced Industrial Science & Technology, Tsukuba, Japan; 3 Peking Union Medical College, Beijing, China; 4 University of Tsukuba, Ibaraki, Japan

**Keywords:** propolis, CAPE, γCD, complex, stable, anticancer

## Abstract

Besides honey, honeybees make a sticky substance (called propolis/bee glue) by mixing saliva with poplar tree resin and other botanical sources. It is known to be rich in bioactivities of which the anticancer activity is most studied. Caffeic acid phenethyl ester (CAPE) is a key anticancer component in New Zealand propolis. We have earlier investigated the molecular mechanism of anticancer activity in CAPE and reported that it activates DNA damage signaling in cancer cells. CAPE-induced growth arrest of cells was mediated by downregulation of mortalin and activation of p53 tumor suppressor protein. When antitumor and antimetastasis activities of CAPE were examined in vitro and in vivo, we failed to find significant activities, which was contrary to our expectations. On careful examination, it was revealed that CAPE is unstable and rather gets easily degraded into caffeic acid by secreted esterases. Interestingly, when CAPE was complexed with γ-cyclodextrin (γCD) the activities were significantly enhanced. In the present study, we report that the CAPE-γCD complex with higher cytotoxicity to a wide range of cancer cells is stable in acidic milieu and therefore recommended as an anticancer amalgam. We also report a method for preparation of stable and less-pungent powder of propolis that could be conveniently used for health and therapeutic benefits.

## Introduction

Cancer is a complex disorder involving abnormal cell growth and with a potential to invade or spread to other parts of the body. Cancerous cells lose their normal control on cell division and develop into unwanted masses of cells called tumors that often become malignant and invade other parts of the body through the blood or lymph system by a process called metastasis. In contrast to the normal cells that divide, differentiate, and finally mature into distinct cell types with specific functions, cancer cells dedifferentiate and specialize to divide uncontrollably. Whereas normal cells respond to intra- and extracellular growth regulatory signals such as program cell death or apoptosis, cancer cells evolve mechanism(s) that override these controls and the normal immune system responses. Furthermore, cancer cells or tissues directly affect the surrounding normal cells or tissues, blood vessels from which they get nutrients and oxygen supply.^1^ Paralleling the complex nature of cancer, its etiology has been ascribed to a multitude of changes at the genetic level, lifestyle factors, food, and environmental conditions.^2-9^

There are 3 major approaches to treat cancer, that is, surgical excision, irradiation, and chemotherapy. The comparative value of these approaches depends on tumor type and stage of cancer. The major therapeutic approach for the treatment of benign and metastasized cancer is chemotherapy; however, this treatment suffers from several limitations including (1) most chemotherapeutic drugs lack selectivity toward cancer cells and hence result in severe toxicity and side effects^10,11^ and (2) P-glycoproteins in the cancer cells activate and mediate multidrug resistance in malignant cells.^12,13^ Heterogeneous cell populations in individual cancers or different tumors also give rise to a variety of drug-resistant cancer stem cells^14^ that contribute to tumor relapse. Zimmerman et al^15^ have described the limited aqueous solubility of plant-derived anticancer drugs as a hurdle to their effective use. These are often hydrophobic in nature and require different solvents to formulate the dosage that also generate severe toxicity. Hence, it is extremely important to design NEW (natural, efficient, and welfare) drugs with additional useful characteristics including cost-effectiveness and targeted delivery. Discovery of NEW selectively targeting drugs is still slow and has high failure rate, particularly in the advanced stages of cancer.^11,16^

Honey and propolis have been shown to possess beneficial activities for human health since ancient times. Propolis is a complex mixture of bee secretions and plant-derived compounds mixed together and is used by bees to build their hives. In general, raw propolis is composed of around 50% resins, 30% waxes, 10% essential oils, 5% pollen, and 5% of various organic compounds.^17^ More than 300 constituents have been identified in bee propolis.^18^ The proportion of various substances present in the propolis depends on its place and time of collection. Traditionally, Egyptians used bee-glue to embalm their cadavers, because of its putrefactive properties. Greek and Roman physicians used bee-glue as an antiseptic and healing product in wound treatment, especially prescribed for topical therapy of cutaneous and mucosal wounds.^19^ Antibacterial usage of propolis became very popular in Europe between the 17th and 20th centuries. In the late 19th century, propolis was widely used for its healing properties. It was also used in Soviet clinics to treat tuberculosis during the Second World War. Currently, propolis is widely considered as a natural remedy and healing reagent. Because of its antimicrobial, antiviral, and antioxidant properties, it is popular in cosmetics and alternative home medicine for various diseases including cold syndrome (respiratory tract infections, common cold, and flu), wound healing, burns, acne, herpes genitalis, and neurodermatitis. It is a common ingredient in commercial preparations for mouthwash and toothpaste, to prevent caries and to treat gingivitis and stomatitis, and it is commercially available in the form of capsules, creams, throat lozenges, and powder.

Many analytical methods have been described to separate and identify the constituents of propolis, which include benzoic acids and derivatives, polyphenols and flavonoids, cinnamic alcohol, cinnamic acid and their derivatives including terpene and sesquiterpene alcohols, benzaldehyde derivatives, amino and other acids and their derivatives, aliphatic and heteroaromatic hydrocarbons, minerals, and sugars.^19,20^ Constituents of propolis are determined by its origin, that is, specific flora of the region. Whereas New Zealand propolis possesses CAPE (caffeic acid phenethyl ester) as a main component, Brazilian green propolis contains artepillin C as a main component. Although both kinds of propolis have been demonstrated to possess similar activities, CAPE has been shown to possess pronounced antiproliferative, proapoptotic, antimicrobial, and antioxidative activities.^21-25^ We have recently shown that the CAPE possesses anticancer and antimetastasis activities, and its complex with γ-cyclodextrin (γCD) further enhances anticancer potential.^26^ In the present study, we report that CAPE is effective for a variety of cell lines. It is an essential anticancer component of propolis and could be stabilized by γCD in acidic milieu that mimics the intestinal microenvironment. Propolis with high content of CAPE and its complex with γCD may be suitable for cancer treatment. We also report a method for the preparation of propolis-γCD powder that is more stable and less pungent in taste, and hence is recommended as a user-friendly NEW anticancer amalgam.

## Materials and Methods

### CAPE-γCD Complex

CAPE was purchased from SynphaTec Japan Co, Ltd (Osaka, Japan). A solution of CAPE (98%) and ethanol (96%; equal molar ratio) was gradually added to an aqueous solution of γCD at 25°C. The mixture was continuously stirred for 20 hours following which CAPE-γCD precipitate was generated. The supernatant was removed by centrifugation. Crude CAPE-γCD was washed with water and chloroform followed by drying in vacuo. A solution of New Zealand propolis ethanol extract was gradually added to an aqueous solution of γCD at 25°C. The mixture was homogenized for 1 hour using an Ultra-Turrax homogenizer (IKA-Werke, Staufen im Breisgau, Germany) at 25°C. The homogenized mixture was frozen and freeze-dried. Propolis-γCD complex powder was obtained after grinding the freeze-dried mixture.

### Cell Culture, Treatments, and Proliferation Assays

Human cancer cells, SKOV3 (ovarian carcinoma), HT1080 (fibrosarcoma), A549 (lung carcinoma), HeLa (cervical carcinoma), U2OS (osteosarcoma), MCF7 and MDA-MB-231 (breast adenocarcinoma; ER positive and triple negative, respectively), and IMR32 (neuroblastoma) cells were cultured in Dulbecco’s modified Eagle’s medium (Gibco BRL, Grand Island, NY) supplemented with 10% fetal bovine serum at 37°C in a humidified incubator set at 5% CO_2_ and 95% air. CAPE and CAPE-γCD complex were dissolved in dimethylsulfoxide to make 1 mM stocks and added to the complete cell culture medium to obtain the working concentrations as indicated. Morphological observations, crystal violet staining, and cell viability (MTT and colony-forming assays) were determined as described earlier.^26^

### Cytotoxicity Assay

The effect of CAPE and CAPE-γCD on cell viability was determined using quantitative colorimetric assays. After overnight incubation, the cells (5 × 10^3^/well) were treated with CAPE and CAPE-γCD as indicated. Vital dye, MTT (0.5 mg/mL) was added to the cell culture medium at the end of treatments and placed in a humidified incubator (37°C and 5% CO_2_) for 4 hours. MTT-containing medium was replaced with dimethylsulfoxide (100 µL) to dissolve purple formazan crystals. Absorbance was measured at 550 nm using spectrophotometer (TECAN, Männedorf, Switzerland). Experiments were done in triplicate. The standard deviation and statistical significance of the data were determined by unpaired *t* test using GraphPad software.

### Morphological Observations

The cells were cultured in 12-well plates, and on reaching 60% confluency, they were treated with different concentrations of CAPE, CAPE-γCD, and γCD. After 48 to 72 hours, morphological changes were recorded under a phase contrast microscope.

### In Vivo Antitumor Assays

The tumor-inhibitory effect of CAPE and propolis was examined using nude mice subcutaneous xenografts. Balb/c nude mice (4 weeks old, female) were bought from Nihon Clea (Tokyo, Japan). Animals were acclimatized in the laboratory for 1 week. Cells were injected subcutaneously (2.5 to 5.0 × 10^6^ suspended in 0.2 mL of growth medium) into the abdomen of nude mice. Either CAPE (200 mg/kg body weight) or propolis (250 mg/kg body weight) was administered by oral route every alternate day starting 1 day after injection of cells. Tumor formation and body weight of mice were monitored every alternate day. Volume of the subcutaneous tumors was calculated as *V* = *L* × *W*^2^/2, where *L* was length and *W* was the width of the tumor, respectively. Statistical significance of the data was calculated from 3 independent experiments (n = 3 per experiment). All the procedures were carried out in accordance with the Animal Experiment and Ethics Committee, Safety and Environment Management Division, National Institute of Advanced Industrial Science & Technology, Tsukuba, Japan.

## Results and Discussion

**Figure 1.**
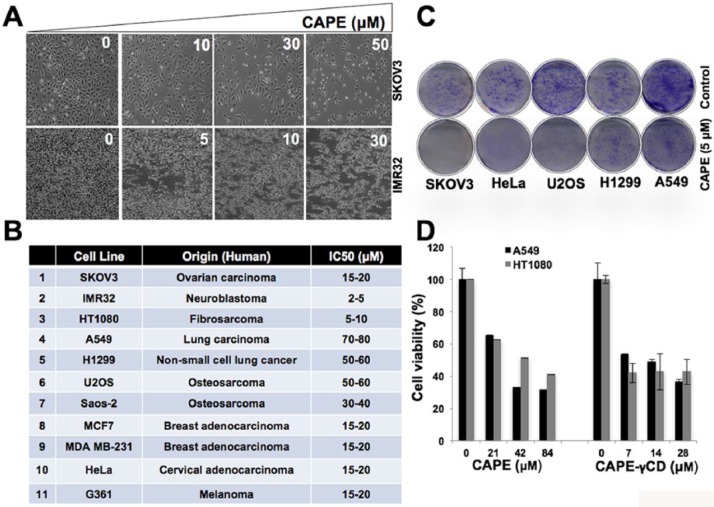
CAPE (caffeic acid phenethyl ester) is cytotoxic to a variety of human cancer cells. (A) Morphology of human cancer (SKOV3 and IMR32) cells treated with increasing doses of CAPE. (B) IC50 for a variety of human cancer cells is shown. (C) Effect of CAPE (5 µM) on human cancer cells in long-term viability assays. (D) Cytotoxicity of CAPE and CAPE-γCD (cyclodextrin) conjugate showing significantly higher effect of the latter.

We had earlier performed cDNA array on control and CAPE-treated breast cancer cells and reported an activation of DNA damage signaling, involving upregulation of GADD45α and p53 tumor suppressor proteins in CAPE-treated cells. Bioinformatics and molecular docking analyses revealed that CAPE disrupts mortalin-p53 complexes.^26^ We provided experimental evidence and demonstrated that CAPE-induced disruption of mortalin-p53 complexes leads to nuclear translocation and activation of p53 resulting in growth arrest in cancer cells. Furthermore, CAPE-treated cells exhibited downregulation of mortalin and several other key regulators of cell migration accounting for its antimetastasis activity.^26^ Since mortalin is enriched in a variety of cancer cell lines and has been suggested as an anticancer target, we examined the effect of CAPE in a variety of cancer cells. As shown in Figure 1, we found that CAPE was cytotoxic to a variety of cancer cells. Although its IC50 ranged from 5 to 80 µM in typical cell viability assays performed with 48-hour incubation (Figure 1A and B), long-term viability assays revealed that 5 µM CAPE caused significant reduction in colony forming efficacy in a variety of cancer cells (Figure 1C and data not shown). Furthermore, CAPE-γCD conjugate showed higher cytotoxicity as compared with CAPE alone (Figure 1D).

**Figure 2.**
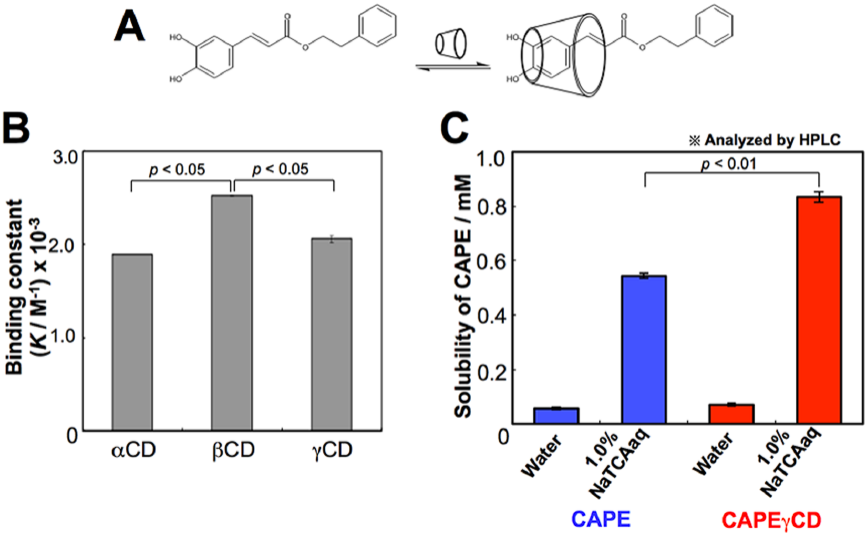
CAPE-γCD (caffeic acid phenethyl ester–γ-cyclodextrin) conjugate is stable in acidic environment. (A) Structure of CAPE and its conjugate with γCD is shown. (B) Binding constants of CAPE with α-, β-, and γ-CD are shown. (C) Solubility of CAPE in water and 1.0% taurocholic acid solution as determined by high-performance liquid chromatography analysis is shown.

We had earlier reported that CAPE, by itself, is unstable but in complex with γCD becomes stable.^26^ We, in the present study, prepared CAPE-CD complex. Binding constants of CAPE with α-, β-, and γ-CD were determined by UV/Vis (ultraviolet–visible) spectroscopic titration method. Similar *K* values (2 × 10^3^ M^−1^) were obtained for the complexation of CAPE alone and with CDs (Figure 2A and B). Next, we examined the solubility of CAPE and CAPE-γCD complex in 1.0% taurocholic acid solution that mimicked intestinal environment. As shown in Figure 2C, CAPE-γCD showed higher solubility than CAPE alone in 1.0% taurocholic acid solution, which endorsed its use in vivo. This may account for the higher tumor suppressor activity of CAPE-γCD complex as compared with CAPE in vivo as reported in our earlier study.^26^

**Figure 3.**
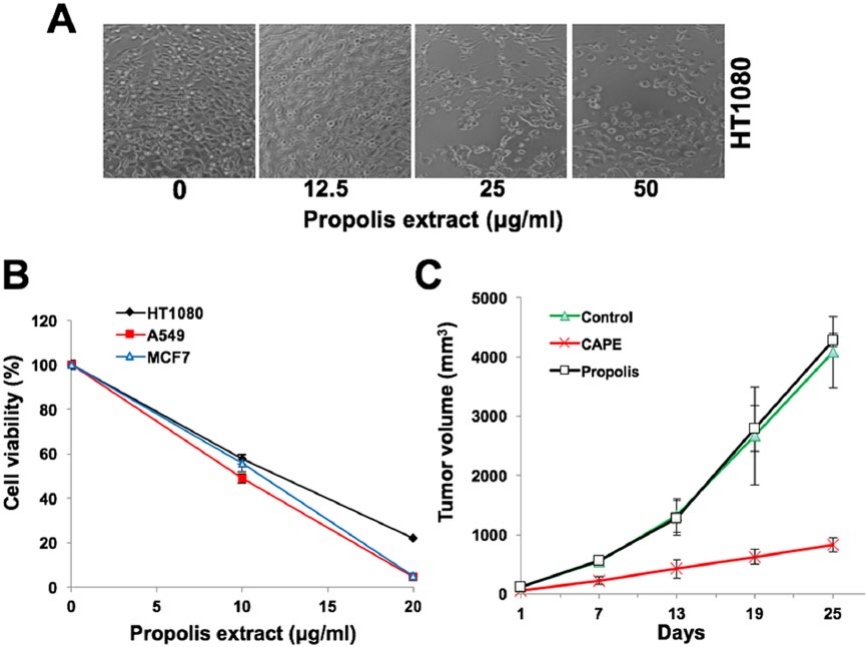
Effect of ethanol extract of propolis on in vitro and in vivo growth of human fibrosarcoma (HT1080). (A) Cells treated with increasing doses of propolis extract are shown. (B) IC50 as determined by MTT assays was 10 to 15 µg/mL. (C) Nude mice tumor progression assay of HT1080 cells in subcutaneous xenografts showed tumor suppression by CAPE (caffeic acid phenethyl ester), but not with propolis that possessed about 1.7% CAPE.

We next prepared an ethanol extract of propolis and examined its cytotoxicity to cancer cells in vitro and in vivo. As shown, it showed dose-dependent cytotoxicity in the range of 10 to 25 µg/mL to all cancer cells tested (Figure 3A and B). However, in vivo tumor formation assays revealed no effect on the growth of HT1080 tumors in subcutaneous xenografts (Figure 3C). We performed high-performance liquid chromatography analysis of the propolis extract and found that it contained low level (1.7%) of CAPE in contrast to 5% to 7% usually found in propolis extract. Although propolis extract with low content of CAPE was cytotoxic to cancer cells, it was ineffective for tumor suppression activity in nude mouse assays (Figure 3B and C). These data suggested that CAPE is an essential component of propolis, responsible for its anticancer activity in vivo. Propolis extracts with moderate levels of CAPE (~5%) should be further considered for cancer treatment in appropriate experimental models.

**Figure 4.**
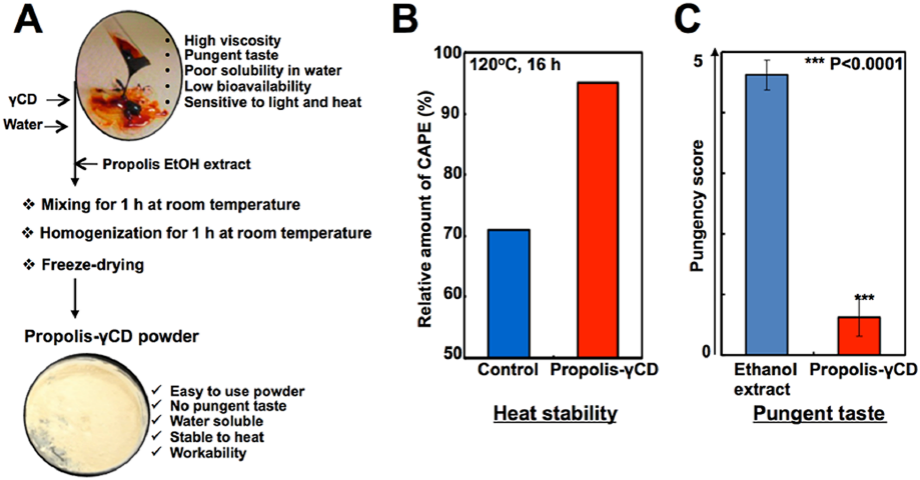
Preparation of propolis powder. (A) Schematic flow showing the method for preparation of propolis-γCD (cyclodextrin) powder is shown. Characterization of propolis-γCD powder exhibited high heat stability (B) and less pungent taste (C) as compared with the propolis alone.

Propolis has been shown to contain high amounts of polyphenols such as flavonoids and caffeic acid derivatives^27^ that are very sensitive to light, heat, and oxidation^28^ and undergo degradation.^26^ We have earlier reported that the stability and bioavailability of CAPE could be enhanced by its complex with γCD. In nude mouse tumor progression assays using subcutaneous xenografts of human fibrosarcoma, tumors showed significantly delayed progression in CAPE and CAPE-γCD fed mice.^26^ Furthermore, CAPE-γCD fed mice showed stronger suppression of tumor growth than the CAPE group. Based on the present information and the fact that CDs are used in food for taste masking and to increase the bioavailability of active components of functional food,^28-30^ propolis-γCD complex was considered. Such a complex (prepared by mixing of ethanol extract of propolis with γCD; Figure 4A) showed high stability to heat (Figure 4B) and possessed less pungent taste (Figure 4C). Furthermore, whereas propolis possessed high viscosity and poor solubility in water, white or light cream powder of propolis-γCD dispersed well in water (Figure 4A). Antitumor efficacy of propolis-γCD complex in a variety of tumor models warrant further studies.

Propolis is a well-known health supplement that is extremely popular in Australia and New Zealand. It is constantly marketed in Japan with sales exceeding US$300 million/year. It is known for a variety of effects of which anticancer is well established by laboratory studies.^21-27^ Some unfavorable characteristics of propolis include high viscosity, pungent taste, poor solubility in water, sensitivity to light and heat because of high polyphenol content, and low bioavailability. Furthermore, CAPE, a major anticancer bioactive in propolis, has been reported to be heat-sensitive and easily degradable. With the use of CDs, we generated thermostable CAPE-γCD as well as propolis-γCD complexes that may be further investigated for use (either as functional food or medicine) in treatment of cancer and other ailments.
